# Inpatient initiation of long-acting injectable buprenorphine for adolescents and young adults with opioid use disorder

**DOI:** 10.1016/j.josat.2026.210008

**Published:** 2026-04-30

**Authors:** Jayme L. Congdon, Alexander R. Bazazi, Maria M. Rossi, Monica Stemmle, Annie Chang, Sherman Lau, Lee A. Trope

**Affiliations:** a Department of Pediatrics and Philip R. Lee Institute for Health Policy Studies, University of California, San Francisco, 490 Illinois St, San Francisco, CA, 94143, United States; b Department of Psychiatry and Behavioral Sciences, University of California, San Francisco, 1001 Potrero Ave #7M2, San Francisco, CA, 94110, United States; c Burrell College of Osteopathic Medicine, 3501 Arrowhead Dr, Las Cruces, NM, 88001, United States; d Pediatrics Department, Santa Clara Valley Medical Center, 751 S Bascom Ave, San Jose, CA, 95128, United States; e Family Medicine Department, Valley Homeless Healthcare Program, Santa Clara Valley Healthcare, 751 S Bascom Ave, San Jose, CA, 95128, United States; f Pharmacy Department, Santa Clara Valley Medical Center, 751 S Bascom Ave, San Jose, CA, 95128, United States

**Keywords:** Opioid use disorder, Opioid addiction, Substance use disorder, Medication-assisted treatment, Buprenorphine, Adolescent medicine, Pediatrics, Hospital medicine

## Abstract

**Introduction:**

Long-acting injectable buprenorphine may be a promising treatment option for adolescents and young adults with opioid use disorder (OUD). However, little is known about its safety, tolerability, and effectiveness in this age group.

**Methods:**

We conducted a 1-year feasibility study of elective inpatient buprenorphine induction followed by administration of long-acting injectable buprenorphine and linkage to ongoing substance use care in individuals <21 years in a safety-net health system (*n* = 18).

**Results:**

Eighteen of 22 (82%) of patients admitted to an inpatient unit for buprenorphine initiation opted to receive the long-acting injectable formulation upon completion of induction. Of 18 patients who received long-acting injectable buprenorphine, 15 (83%) and 12 (67%) remained in care at one- and two-months post discharge, respectively. Eleven (61%) participants received repeat doses at both one- and two-months post discharge. Fourteen (78%) and 12 (67%) participants had not returned to opioid use at one- and two- months post discharge, respectively.

**Conclusion:**

The findings from this study of adolescents and young adults with OUD demonstrated that inpatient initiation of long-acting injectable buprenorphine after a brief induction with short-acting buprenorphine may be safe, well tolerated, and effective in terms of linkage, retention in care, and return to opioid use.

## 1. Introduction

Opioid use disorder (OUD) continues to be a leading threat to the health and lives of U.S. adolescents. Treatment of OUD is critical for reducing the downstream risk of all-cause and opioid-related morbidity and mortality (Sordo et al., 2017). Medication treatment for OUD (MOUD) is the gold-standard, based on robust evidence in adults (Santo Jr et al., 2021) and a growing body of evidence in adolescents (Becker et al., 2022; Borodovsky et al., 2018; Camenga et al., 2019). The three medication options for treating OUD include buprenorphine, methadone, and naltrexone. Of these, only buprenorphine labeling specifies use in individuals <18 years. Despite its availability since 2002 and recommendations for its use by the American Academy of Pediatrics and the American Society of Addiction Medicine (American Academy of Pediatrics Committee on Substance Use and Prevention, 2016; “The ASAM National Practice Guideline for the Treatment of Opioid Use Disorder,” 2020), buprenorphine remains severely underutilized– the most recent dispensing data showed that only about 5% of adolescents and young adults with OUD received buprenorphine (Lee et al., 2025). Beyond the barriers to initiating treatment, adolescents who start buprenorphine have relatively poor retention in care. Younger age was consistently associated with worse retention and shorter treatment duration in a review of OUD treatment outcomes in young adults (Fishman et al., 2024). Adolescents face numerous patient, system, and policy level barriers to continuing treatment with buprenorphine, for example developmental factors that impede adherence to daily dosing regimens and frequent medical appointments, stigma among social networks and clinicians, medication access difficulties at pharmacies, and state confidentiality and consent laws (Camenga et al., 2019; English & Gudeman, 2024; Fishman et al., 2024; Herrera et al., 2024; Raney et al., 2025).

A monthly long-acting injectable form of buprenorphine (LAIB), labeled for individuals ≥18 years, became available in 2017. LAIB has the potential to mitigate some key barriers to adolescent MOUD treatment (Calihan & Bagley, 2024). Studies of LAIB in adults have demonstrated safety, tolerability, and clinical effectiveness, with improvement in outcomes such as retention in care, return to use, overdose, and utilization of emergency department and inpatient services (Andorn et al., 2020; Frost et al., 2019; Haight et al., 2019; Ochalek et al., 2023; Wenzel et al., 2021; Yarborough et al., 2024). While LAIB seems a promising treatment option for adolescents younger than 18 years as well, publications describing its use in this age group are limited to case reports and small case series (Allami et al., 2024; Azar et al., 2020; Neptune & Kaliamurthy, 2024; Schmuhl et al., 2024; Velagapudi et al., 2024). Developing age-appropriate clinical guidance requires larger studies in pediatric patients, for example to guide care around factors such as caregiver consent, developmentally tailored patient and caregiver education, and age-appropriate follow-up plans.

One potential barrier to the broader use of LAIB across all age groups was the preexisting label instructions recommending patients initiate short-acting buprenorphine and maintain a stable daily dose for seven days prior to transitioning to LAIB. In February of 2025, the U.S. Food and Drug Administration approved manufacturer label changes that include a rapid initiation protocol with administration after only one dose of sublingual buprenorphine and one-hour of observation (Indivior UK Limited, 2025). Consistent with this change, several reports describe safely initiating LAIB after an abbreviated induction with short-acting buprenorphine in adults (O’Conor et al., 2024; Wenzel et al., 2021) and one adolescent (Azar et al., 2020) in an inpatient setting and in a large sample of adults in emergency departments (D’Onofrio et al., 2023).

The lack of published studies on the safety, tolerability, and effectiveness of LAIB in adolescents and young adults is a critical gap, given persistently high rates of OUD and poor access to MOUD initiation and maintenance. To address this gap, here we describe a feasibility study of brief buprenorphine induction and initiation of LAIB in adolescents with OUD in a safety-net hospital.

## 2. Methods

From March 2024–March 2025, we conducted a feasibility study of inpatient administration of LAIB immediately following buprenorphine induction in an inpatient pediatric unit.

### 2.1. Intervention overview

**Fig. 1.**
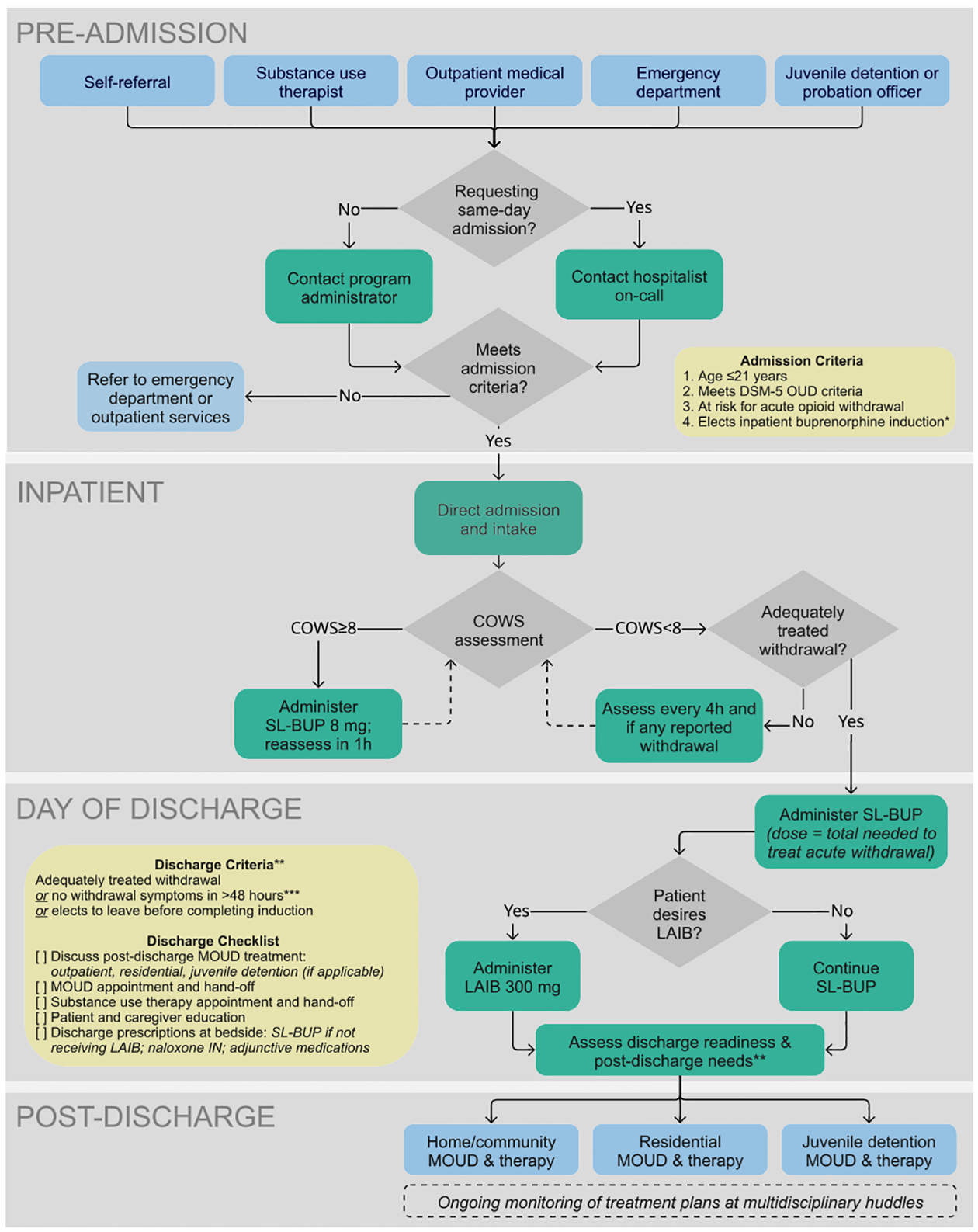
Buprenorphine initiation protocol for the inpatient pediatric unit at Santa Clara Valley Medical Center, San Jose, California (n = 18). COW-S=Clinical Opiate Withdrawal Scale (Wesson & Ling, 2003), LAIB = long-acting injectable buprenorphine, MOUD = Medication for Opioid Use Disorder, naloxone IN = naloxone intranasal, SL-BUP = buprenorphine and naloxone sublingual film; *Pediatric hospitalists and program administrator discuss with referred patients available options for initiating treatment (i.e., inpatient, outpatient, emergency department); **Discharge planning includes shared decision-making with patient/caregiver and clinical team; ***Offer BUP-NLX 8 mg daily as maintenance dose and complete discharge checklist.

This intervention took place within a public safety-net hospital system in California. The pediatric hospitalist team began admitting adolescents and young adults to the 40-bed inpatient pediatric unit for elective buprenorphine induction in 2021. Building on the previously published inpatient buprenorphine induction protocol, in 2024 LAIB was added to the hospital formulary and patients could opt to receive Sublocade^®^ 300 mg (monthly buprenorphine extended-release injection) after completing brief induction with sublingual buprenorphine (Trope et al., 2023). When counseling youth about options for buprenorphine maintenance, hospitalists discussed the risks and benefits of short-acting sublingual buprenorphine and LAIB, including effectiveness, dosing interval, route of administration, potential for misuse, and potential side effects (e.g. injection-site reactions). The inpatient buprenorphine induction protocol in Fig. 1 includes: admission criteria, direct admission to the inpatient pediatric unit, monitoring and treatment of withdrawal with sublingual buprenorphine, optional LAIB before discharge, post-discharge care coordination, and discharge criteria. Clinicians used shared decision-making with patients to determine the timing of LAIB administration, with the goal of balancing length of stay while minimizing patient withdrawal symptoms. Prior to discharge, the inpatient team arranged for patients to continue treatment with an outpatient MOUD provider and therapist, at a residential treatment facility, or in juvenile detention, if applicable.

### 2.2. Setting and participants

Participants were ≤21 years, met DSM-5 criteria for OUD, and elected inpatient buprenorphine induction. Analyses included patients who elected to receive LAIB prior to discharge and were admitted and eligible for their 2-month follow-up appointment and LAIB dose within the 1-year study period (*n* = 18). For patients admitted more than once during the study period (n = 1), we included only data from the first admission. The Santa Clara Valley Medical Center Institutional Review Board reviewed and approved these procedures and determined that the analysis of deidentified program evaluation data met criteria for exempt status.

### 2.3. Data collection and analysis

Demographics (age, gender, race, insurance type) were from the electronic health record (EHR). Clinical characteristics at induction (urine toxicology, buprenorphine induction dose, discharge disposition, and length of stay) were from the EHR, including chart notes, medication records, and nursing documentation. Clinical outcomes at 1 and 2 months (retention in care, repeat LAIB doses, return to opioid use) were from chart review, phone follow-up calls with patients, outpatient clinic providers, and a statewide prescription drug monitoring database. Return to use data was from urine toxicology at the MOUD clinic (11/18 at 1 month, 8/18 at 2 months); if urine toxicology was unavailable, return to use was by patient self-report (2/18 at 1 and 2 months) or outpatient provider report (3/18 at 1 month, 4/18 at 2 months). We conducted descriptive statistical analysis of demographic and clinical data. Data on serious adverse events (i.e., respiratory depression, injection site reactions, adrenal insufficiency, hypersensitivity reactions, precipitated withdrawal, orthostatic hypotension) and overdose was from the EHR and follow-up phone calls with patients and outpatient clinic providers (Indivior UK Limited, 2025).

## 3. Results

Between March 2024 and March 2025, 18 of 22 (82%) patients admitted to our pediatric unit for buprenorphine induction elected to receive LAIB before discharge. Patients who received LAIB were 16 to 20 years (mean 17.9, SD 1.2) and 72% female. Patients identified as Latinx (13/18, 72%) or White (5/18, 28%). Most patients had Medicaid insurance (13/18, 72%; Table 1).

Admission urine toxicology detected fentanyl (16/18, 89%), opiates (1/18, 6%), amphetamine/methamphetamine (6/18, 33%), and cocaine (3/18, 17%). One patient admitted for buprenorphine withdrawal had a urine toxicology screen that was positive only for buprenorphine. Patients received 8–56 mg of sublingual buprenorphine during induction (mean 24.4, SD 11.8). LAIB administration occurred between 9 and 55 h after the first dose of sublingual buprenorphine (mean 24.4, SD 11.8). Total length of stay was 19–66 h (mean 42.1, SD 15.2).

Fifteen (83%) and 12 (67%) of 18 patients remained in substance use care at one- and two-months post discharge, respectively. Eleven (61%) patients received repeat doses of LAIB at both one- and two-months post discharge. The majority of patients did not return to opioid use at one month (14/18, 78%) or two months (12/18, 67%).

No cases of precipitated withdrawal occurred. One serious adverse event occurred in a patient who developed skin necrosis at the LAIB injection site, which resolved with wound care and without need for surgical extraction of the depot; this patient subsequently elected to switch to a different LAIB product (i.e., Brixadi^®^).

## 4. Discussion

This interventional study of adolescents and young adults with OUD provides evidence that inpatient initiation of LAIB after a brief induction with short-acting buprenorphine was overall safe and well tolerated. Most youth remained in care and did not return to opioid use. These LAIB safety and tolerability findings in 18 adolescents are consistent with smaller published case reports and series of adolescents, most of which also found similarly favorable treatment outcomes and no cases of precipitated withdrawal (Azar et al., 2020; Schmuhl et al., 2024; Velagapudi et al., 2024; Wenzel et al., 2021). The success of our LAIB induction protocol also aligns with the update to the Sublocade^®^ manufacturer label to include a rapid transition from sublingual buprenorphine to LAIB (Indivior UK Limited, 2025). Taken together with other reports describing LAIB initiation in adolescents, these findings provide preliminary evidence for the safety and effectiveness of LAIB in this age group (Allami et al., 2024; Azar et al., 2020; Neptune & Kaliamurthy, 2024; Schmuhl et al., 2024; Velagapudi et al., 2024).

The majority of patients here were linked to outpatient treatment, which was similar to our experience with inpatient initiation of short-acting buprenorphine in adolescents and higher than in a study of inpatient LAIB initiation in adults (O’Conor et al., 2024; Trope et al., 2023). Successful linkage between the hospital and outpatient clinics supports further exploration of clinical models that “meet patients where they are” for treatment initiation, such as inpatient and emergency department settings, which require establishing care in another setting for ongoing treatment (Herring et al., 2024; Trope et al., 2023). Future studies assessing the effectiveness, feasibility, and acceptability of initiating LAIB in pediatric patients in these various settings would provide critical information to guide clinical programs and related policies. The present study design did not allow for assessment of the impact of care coordination on follow-up outcomes, though future implementation studies should consider the influence of such support.

We found that 18 of 22 youth in our program opted to receive LAIB rather than continuing short-acting sublingual buprenorphine, and many followed up to receive their second and third doses, suggesting high interest in the LAIB option. Future research should assess the generalizability of these findings and explore the factors underlying patient preferences to ensure that clinical services and pharmacy formularies align. Patient preferences should also inform payer formularies and policies, with consideration of adding coverage and removing prior authorization requirements, which are known barriers to LAIB access (Andraka-Christou et al., 2023).

The participants in this intervention were all electively admitted specifically for opioid withdrawal and initiation of buprenorphine treatment. Beyond elective inductions, the inpatient setting may have a role in initiating treatment in patients admitted for nonfatal overdoses or in patients who develop opioid withdrawal while admitted for non-opioid related indications. These scenarios support the need for hospitalists to be trained and comfortable prescribing buprenorphine (Camenga & Barelli, 2023).

The small sample size limits the generalizability of these findings, though this evaluation provides useful preliminary data to inform future larger studies. Additionally, this study took place in a safety-net health system that primarily serves low-income, publicly insured patients. Therefore, research in other settings and with distinct patient populations is essential for assessing clinical and implementation outcomes of similar protocols for LAIB in youth.

In conclusion, this study provides preliminary evidence that starting LAIB after a brief inpatient buprenorphine induction may be safe, well tolerated, and effective in terms of linkage, retention, and return to use. Future research should continue to assess the safety, effectiveness, feasibility, and acceptability of LAIB as a promising addition to the menu of OUD treatment options. Such research would provide critical input into institutional, payer, legislative, and administrative policies to promote improved access and outcomes for adolescents and young adults with OUD.

## Figures and Tables

**Table 1:** Characteristics and clinical outcomes of adolescents who initiated long-acting injectable buprenorphine in San Jose, California in 2024–25 (*n* = 18).

	N (%) or Range, Mean (SD)
**Demographics**	
Age, years	16–20, 17.9 (1.2)
<18	7 (39%)
≥18	11 (61%)
Gender	
Female	13 (72%)
Male	5 (28%)
Race and ethnicity	
Latino/a/e	13 (72%)
White	5 (28%)
Insurance type	
California Medicaid	16 (89%)
Commercial	2 (11%)
**Clinical characteristics**	
Urine toxicology	
Amphetamine/methamphetamine	6 (33%)
Benzodiazepines	4 (22%)
Cocaine	3 (17%)
Fentanyl	16 (89%)
Opiates	1 (6%)
SL-BUP dose for induction, mg	8–56, 24.4 (11.8)
Weight, kg	40.8–81.6, 56.1 (11.2)
Discharge disposition	
Home	15 (83%)
Juvenile detention	2 (11%)
Residential treatment facility	1 (6%)
Time between first SL-BUP dose and LAIB dose, hours	9–55, 24.2 (10.7)
Length of stay, hours	19–66, 42.1 (15.2)
**Clinical outcomes**	
Retention in any substance use care at 1 month	15 (83%)
MOUD clinic	14 (78%)
Substance use therapy	6 (33%)
Retention in any substance use care at 2 months	12 (67%)
MOUD clinic	12 (67%)
Substance use therapy	6 (33%)
Received LAIB at 1 month	11 (61%)
Received LAIB at 2 months	11 (61%)
Returned to opioid use at 1 month	
No	14 (78%)
Yes	2 (11%)
Lost to follow-up	2 (11%)
Returned to opioid use at 2 months	
No	12 (67%)
Yes	2 (11%)
Lost to follow-up	4 (22%)
Serious adverse event^a^	1 (6%)

LAIB = long-acting injectable buprenorphine, SL-BUP = buprenorphine and naloxone sublingual film.

a One patient experienced skin necrosis at the LAIB injection site, which resolved with wound care and without need for surgical extraction of the depot.

## References

[R1] Allami N, O’Connor K, & Bagley SM (2024). A case of severe opioid and methamphetamine use disorder in a 14 year old. Addiction Science & Clinical Practice, 19(1), 54. 10.1186/s13722-024-00487-1 39030629 PMC11264861

[R2] American Academy of Pediatrics Committee on Substance Use and Prevention. (2016). Medication-assisted treatment of adolescents with opioid use disorders. Pediatrics, 138(3), Article e20161893. 10.1542/peds.2016-1893 27550978

[R3] Andorn AC, Haight BR, Shinde S, Fudala PJ, Zhao Y, Heidbreder C, … Rutrick D (2020). Treating opioid use disorder with a monthly subcutaneous buprenorphine depot injection: 12-Month safety, tolerability, and efficacy analysis. Journal of Clinical Psychopharmacology, 40(3), 231–239. 10.1097/JCP.0000000000001195 32282418 PMC7188268

[R4] Andraka-Christou B, Simon KI, Bradford WD, & Nguyen T (2023). Buprenorphine treatment for opioid use disorder: comparison of insurance restrictions, 2017–21. Health Affairs, 42(5), 658–664. 10.1377/hlthaff.2022.01513 37126752 PMC10275692

[R5] Azar P, Wong JSH, Jassemi S, Moore E, Vo DX, Nikoo M, & Young S (2020). A case report: Rapid micro-induction of buprenorphine/naloxone to administer buprenorphine extended-release in an adolescent with severe opioid use disorder. The American Journal on Addictions, 29(6), 531–535. 10.1111/ajad.13050 32346944

[R6] Becker SJ, Scott K, Helseth SA, Danko KJ, Balk EM, Saldanha IJ, … Steele DW (2022). Effectiveness of medication for opioid use disorders in transition-age youth: A systematic review. Journal of Substance Abuse Treatment, 132, Article 108494. 10.1016/j.jsat.2021.108494 PMC8628023 34098208

[R7] Borodovsky JT, Levy S, Fishman M, & Marsch LA (2018). Buprenorphine treatment for adolescents and young adults with opioid use disorders: A narrative review. Journal of Addiction Medicine, 12(3), 170–183. 10.1097/ADM.0000000000000388 29432333 PMC5970018

[R8] Calihan JB, & Bagley SM (2024). Injectable buprenorphine: An opportunity to improve treatment access for youth with opioid use disorder. Journal of Adolescent Health, 75(1), 13–14. 10.1016/j.jadohealth.2024.04.010 38880557

[R9] Camenga DR, & Barelli P (2023). It is time for pediatric hospitalists to treat opioid use disorder. Hospital Pediatrics, 13(2), e34–e36. 10.1542/hpeds.2022-006940 36683463

[R10] Camenga DR, Colon-Rivera HA, & Muvvala SB (2019). Medications for maintenance treatment of opioid use disorder in adolescents: A narrative review and assessment of clinical benefits and potential risks. Journal of Studies on Alcohol and Drugs, 80(4), 393–402. 10.15288/jsad.2019.80.393 31495374

[R11] D’Onofrio G, Perrone J, Hawk KF, Cowan E, McCormack R, Coupet E, … ED-INNOVATION Investigators. (2023). Early emergency department experience with 7-day extended-release injectable buprenorphine for opioid use disorder. Academic Emergency Medicine: Official Journal of the Society for Academic Emergency Medicine, 30(12), 1264–1271. 10.1111/acem.14782 37501652 PMC10822018

[R12] English A, & Gudeman R (2024). Minor consent and confidentiality a compendium of state and federal laws. http://teenhealthlaw.org/compendium.

[R13] Fishman M, Wenzel K, Gauthier P, Borodovsky J, Murray O, Subramaniam G, … Marsch LA (2024). Engagement, initiation, and retention in medication treatment for opioid use disorder among young adults: A narrative review of challenges and opportunities. Journal of Substance Use and Addiction Treatment, 166, Article 209352. 10.1016/j.josat.2024.209352 PMC11392652 38494051

[R14] Frost M, Bailey GL, Lintzeris N, Strang J, Dunlop A, Nunes EV, … Tiberg F (2019). Long-term safety of a weekly and monthly subcutaneous buprenorphine depot (CAM2038) in the treatment of adult out-patients with opioid use disorder. Addiction (Abingdon, England), 114(8), 1416–1426. 10.1111/add.14636 31013390 PMC6771955

[R15] Haight BR, Learned SM, Laffont CM, Fudala PJ, Zhao Y, Garofalo AS, … Wiest KL (2019). Efficacy and safety of a monthly buprenorphine depot injection for opioid use disorder: A multicentre, randomised, double-blind, placebo-controlled, phase 3 trial. The Lancet, 393(10173), 778–790. 10.1016/S0140-6736(18)32259-1 30792007

[R16] Herrera MC, Darien K, Wood S, Hadland SE, Deanna Wilson J, & Dowshen N (2024). Opportunities to enhance retention on medication for opioid use disorder for adolescents and young adults: Results from a qualitative study with medical providers in Philadelphia, PA. Harm Reduction Journal, 21(1), 210. 10.1186/s12954-024-01113-8 39581981 PMC11587537

[R17] Herring AA, Rosen AD, Samuels EA, Lin C, Speener M, Kaleekal J, … Kalmin MM (2024). Emergency department access to buprenorphine for opioid use disorder. JAMA Network Open, 7(1), Article e2353771. 10.1001/jamanetworkopen.2023.53771 PMC10825722 38285444

[R18] Indivior UK Limited. (2025). Sublocade^®^ (buprenorphine extended-release) package insert. U.S. Food and Drug Administration.

[R19] The ASAM national practice guideline for the treatment of opioid use disorder: 2020 focused update. Journal of Addiction Medicine, 14(2S), (2020), 1. 10.1097/ADM.0000000000000633 32511106

[R20] Lee E, Rikard SM, Guy G Jr., & Terranella A (2025). Trends in buprenorphine dispensing among adolescents and young adults in the US. JAMA, 333(5), 425–427. 10.1001/jama.2024.24121 39714894 PMC11795321

[R21] Neptune A, & Kaliamurthy S (2024). The use of extended-release buprenorphine in the treatment of adolescent opioid use disorder: A case series. Journal of Addiction Medicine. 10.1097/ADM.0000000000001447 39898524

[R22] Ochalek TA, Ringwood KJ, Davis TT, Gal TS, Wills BK, Sabo RT, … Moeller FG (2023). Rapid induction onto extended-release injectable buprenorphine following opioid overdose: A case series. Drug and Alcohol Dependence Reports, 7, Article 100144. 10.1016/j.dadr.2023.100144 PMC10073633 37033158

[R23] O’Conor C, Farhi S, Cowan E, & Fitzgerald R (2024). Inpatient initiation of long-acting injectable buprenorphine at a community hospital: A retrospective case series. Journal of Addictive Diseases, 1–7. 10.1080/10550887.2024.2391145 39219151

[R24] Raney C, Raney J, Tripp P, Halpern-Felsher B, Bruce J, Trope L, & Congdon J (2025). 119. “It’s really hard to just do it by yourself:” Adolescent perspectives on the facilitators, barriers, and health service preferences to initiating and continuing medication treatment for opioid use disorder. Journal of Adolescent Health, 76(3), S61–S62. 10.1016/j.jadohealth.2024.11.135

[R25] Santo T Jr., Clark B, Hickman M, Grebely J, Campbell G, Sordo L, … Degenhardt L (2021). Association of opioid agonist treatment with all-cause mortality and specific causes of death among people with opioid dependence: A systematic review and meta-analysis. JAMA Psychiatry, 78(9), 979–993. 10.1001/jamapsychiatry.2021.0976 34076676 PMC8173472

[R26] Schmuhl KK, Golec A, & Ebersole AM (2024). Early remission of opioid use disorder in an adolescent using buprenorphine extended - Release subcutaneous injection: A case report. The Journal of Adolescent Health, 75(1), 200–202. 10.1016/j.jadohealth.2024.01.012 38402472

[R27] Sordo L, Barrio G, Bravo MJ, Indave BI, Degenhardt L, Wiessing L, … Pastor-Barriuso R (2017). Mortality risk during and after opioid substitution treatment: Systematic review and meta-analysis of cohort studies. BMJ, 357, Article j1550. 10.1136/bmj.j1550 PMC5421454 28446428

[R28] Trope LA, Stemmle M, Chang A, Bashiri N, Bazazi AR, Lightfoot M, & Congdon JL (2023). A novel inpatient buprenorphine induction program for adolescents with opioid use disorder. Hospital Pediatrics, 13(2), e23–e28. 10.1542/hpeds.2022-006864 36683456 PMC12043207

[R29] Velagapudi V, Schuster L, & Sethi R (2024). Buprenorphine: Two adolescent case reports of bridging the transmucosal form to the extended-release subcutaneous injectable form. Journal of Addictive Diseases, 42(4), 426–431. 10.1080/10550887.2023.2251366 38269542

[R30] Wenzel K, Selby V, Wildberger J, Lavorato L, Thomas J, & Fishman M (2021). Choice of extended release medication for OUD in young adults (buprenorphine or naltrexone): A pilot enhancement of the Youth Opioid Recovery Support (YORS) intervention. Journal of Substance Abuse Treatment, 125, Article 108306. 10.1016/j.jsat.2021.108306 34016297

[R31] Wesson DR, & Ling W (2003). The Clinical Opiate Withdrawal Scale (COWS). Journal of Psychoactive Drugs, 35(2), 253–259. 10.1080/02791072.2003.10400007 12924748

[R32] Yarborough BJH, Stumbo SP, Janoff SL, Keast EM, Leo MC, & Leitz SJ (2024). Reduced emergency department use among insured individuals receiving extended-release buprenorphine in a health system setting. Drug and Alcohol Dependence Reports, 11, Article 100233. 10.1016/j.dadr.2024.100233 PMC11063592 38699647

